# Safety and Immunogenicity of the BNT162b2 COVID-19 Vaccine in Immunocompromised Participants 2 Years and Older: Results of an Open-Label Phase 2b Study

**DOI:** 10.3390/vaccines14070602

**Published:** 2026-07-08

**Authors:** Alpana Waghmare, Rucha Dadhe, Robin Kobbe, Lara Danziger-Isakov, Eduardo Sprinz, Flor M. Muñoz, Juleen Gayed, Rohit Solan, Oyeniyi Diya, Bisrat Abraham, Ye Feng, Xia Xu, Todd Belanger, Federico J. Mensa, Roxie Girardin, Özlem Türeci, Uğur Şahin, Kayvon Modjarrad, Kena A. Swanson, Annaliesa S. Anderson, Alejandra Gurtman, Nicholas Kitchin

**Affiliations:** 1 Center for Clinical and Translational Research, Seattle Children’s Research Institute, Vaccine and Infectious Diseases Division, Fred Hutchinson Cancer Center, Seattle, WA 98109, USA; 2 Department of Pediatrics, Division of Infectious Diseases, University of Washington School of Medicine, Seattle, WA 98195, USA; 3 Pfizer Vaccines, Pfizer Ltd., Marlow SL7 1YL, UK; rucha.dadhe@pfizer.com (R.D.);; 4 Institute for Infection Research and Vaccine Development, University Medical Centre Hamburg-Eppendorf, 20246 Hamburg, Germany; 5 Department of Tropical Medicine, Bernhard Nocht Institute for Tropical Medicine and I. Department of Medicine, University Medical Center Hamburg-Eppendorf, 20246 Hamburg, Germany; 6 University of Cincinnati Department of Pediatrics, Cincinnati Children’s Hospital Medical Center, Cincinnati, OH 45229, USA; 7 Infectious Diseases Service, Hospital de Clinicas de Porto Alegre, Porto Alegre 90035-003, Brazil; 8 Department of Pediatrics, Division of Infectious Diseases, Baylor College of Medicine, Houston, TX 77030, USA; 9 Department of Pediatrics, Texas Children’s Hospital, Houston, TX 77030, USA; 10 Pfizer Vaccines, Pfizer Inc., Pearl River, NY 10965, USA; 11 Pfizer Vaccines, Pfizer Inc., Collegeville, PA 19426, USA; 12 Clinical Immunology and High-Throughput Operations, Pfizer Inc., Pearl River, NY 10965, USA; 13 BioNTech, 55131 Mainz, Germany; 14 Viral Vaccines and Immunology, Pfizer Inc., Pearl River, NY 10965, USA

**Keywords:** COVID-19, SARS-CoV-2, immunocompromised, mRNA, vaccine

## Abstract

**Background**: The BNT162b2 vaccine is safe and effective for COVID-19 prevention. BNT162b2 safety and immunogenicity have been evaluated in immunocompromised individuals in real-world observational studies, particularly in pediatric populations, but not in clinical trials. **Methods**: This phase 2b single-arm trial descriptively evaluated a Dose 3 (age-appropriate) BNT162b2 primary series with a Dose 4 in immunocompromised individuals 2–<5, 5−<12, 12–<18, and ≥18 years of age without a previous clinical or microbiological COVID-19 diagnosis. Primary objectives were to describe immune responses, reactogenicity, and adverse events following vaccination. **Results**: Out of 124 participants enrolled, 119 received Dose 3 and 90 received Dose 4. Among participants without evidence of past SARS-CoV-2 infection, neutralizing geometric mean titers (GMTs) and geometric mean fold rises (GMFRs) against the SARS-CoV-2 ancestral strain ranged from 344.6 to 1584.4 and 7.9 to 36.4 at 1 month after Dose 3 and from 1474.0 to 4157.9 and 31.0 to 95.6 at 1 month after Dose 4, respectively, across age groups. Among participants with or without evidence of past infection, GMTs and GMFRs ranged from 787.1 to 2940.6 and 9.6 to 54.3 at 1 month after Dose 3 and from 1031.3 to 13,457.1 and 9.1 to 220.0 at 1 month after Dose 4. Percentages of participants with or without evidence of past SARS-CoV-2 infection achieving seroresponse ranged from 50.0 to 92.9% at 1 month after Dose 3, and from 75.0 to 100% and 33.3 to 100.0% at 1 and 6 months after Dose 4 across age groups, respectively. No new safety signals were identified. **Conclusions**: BNT162b2 was immunogenic, increasing GMTs in immunocompromised individuals ≥2 years old, particularly after Doses 3 and 4. GMT increases were generally similar across age groups and disease subsets. Three or four BNT162b2 doses had a favorable risk-benefit profile in this population.

## 1. Introduction

Immunocompromised individuals, such as organ and hematopoietic cell transplant recipients, those receiving immunomodulator therapy, or those with autoimmune diseases, primary immunodeficiencies, hematologic malignancies, or advanced HIV infection [1], are more susceptible to COVID-19 than healthy individuals [2]. A study of 301 children 1–11 years of age at a tertiary care referral center in 2021, which included 22.3% of children who were SARS-CoV-2 seropositive at baseline, found that 40.5% of 42 immunocompromised patients were seropositive for SARS-CoV-2 antibodies compared with 19.3% of the 259 patients who were not immunocompromised [3]. Although COVID-19 can be mild in immunocompetent children [4,5], immunocompromised individuals are at increased risk for worse outcomes, such as hospitalization, intensive care unit admissions, and death [6,7,8].

The messenger RNA (mRNA)-based COVID-19 vaccine, BNT162b2 (Original, Pfizer-BioNTech), was developed early in the COVID-19 pandemic [9]. In pivotal trials, BNT162b2 was safe and effective against COVID-19 in relation to the ancestral SARS-CoV-2 strain as a two-dose series in healthy individuals ≥16 years of age, adolescents 12–15 years of age, and children 5–11 years of age, and as a three-dose series in healthy children 6 months of age to 4 years of age [10,11,12,13].

Compared with immunocompetent individuals, immunocompromised adults and children generally have a weaker immune response to vaccination due to their condition or the immunosuppressive therapies used to treat their underlying disease [14]. Importantly, immune responses to vaccination vary substantially among individuals with different immunocompromising conditions due to differences in the severity of alterations in the immune system [14]. A systematic review of immunocompromised adults who had received two doses of the COVID-19 vaccine found that organ transplant recipients had the lowest rates of seroconversion, followed by those with hematologic cancers, immune-mediated inflammatory disorders, solid cancers, and HIV infection [15]. An observational study of immunocompromised and healthy children receiving two or three doses of the whole-cell inactivated COVID-19 vaccine (BBIBP-CorV) and/or BNT162b2 demonstrated that most children exhibited immune responses after two doses; however, children who were immunocompromised typically exhibited lower SARS-CoV-2 neutralizing titers than healthy children even after three doses [16]. A prospective cohort study of children 5–11 years of age who had undergone allogeneic hematopoietic stem cell transplantation and received two doses of BNT162b2 found that anti-SARS-CoV-2 antibody response was significantly lower in those on immunosuppressive treatment than in those off treatment at the time of vaccination [17]. In three small studies of children who received a solid organ transplant (SOT), 56 to 73% seroconverted after two doses of BNT162b2 [18,19,20]. Another observational study of immunocompromised participants 12–25 years of age who received two doses of BNT162b2 found significantly lower antibody titers in immunocompromised versus immunocompetent participants [21]. Additionally, rates of breakthrough COVID-19 cases after a two-dose series of BNT162b2 were found to be three times higher in immunocompromised versus immunocompetent individuals [22].

Studies in adults indicate that additional doses of COVID-19 vaccines improve seroresponse rates and antibody titers in immunocompromised populations [15,23,24,25,26,27]. Data on extended BNT162b2 doses, especially in children, are limited. In a study of 37 immunocompromised children and young adults that included SOT recipients, most had detectable humoral response after two doses of an mRNA COVID-19 vaccine, with significantly improved responses after a third dose [28]. Similarly, pediatric SOT recipients showed antibody responses following two doses of an mRNA COVID-19 vaccine, with further improved antibody responses following more than two doses [29]. Herein, we descriptively evaluate the safety, tolerability, and immunogenicity of BNT162b2 following a fourth dose in a phase 2b study in immunocompromised participants ≥2 years of age.

## 2. Methods

### 2.1. Study Design and Participants

This phase 2b, open-label, single-arm study of the original BNT162b2 mRNA-based COVID-19 vaccine in immunocompromised individuals was conducted from October 2021 (first participant’s study visit) to July 2023 (last participant’s study visit) in the United States, Brazil, Germany, and Mexico (NCT04895982 (registration date: 21 May 2021); Eudra CT 2021-001290-23 (date of authority decision: 31 August 2021)). Detailed inclusion and exclusion criteria and study ethical standards are provided in the Supplemental Text. Participants were excluded if they had a past clinical or microbiologic diagnosis of COVID-19; multisystem inflammatory syndrome in children; active graft-vs-host disease, transplant rejection, posttransplant lymphoproliferative disorder, or treatment for these conditions within 3 months before enrollment; or a history of severe adverse reactions associated with a vaccine or severe allergic reaction to any vaccine component. Eligible participants were COVID-19 vaccine-naive and immunocompromised as defined by the criteria in the Supplemental Text.

The study protocol initially included three doses of the original BNT162b2, the first two doses separated by 21 days and the third dose occurring 28 days after Dose 2. The protocol was amended in early 2022 to include a fourth dose of the original BNT162b2 given 3 to 6 months after Dose 3 in line with regulatory authority recommendations. BNT162b2 was administered at 3-μg, 10-μg, and 30-μg dose levels for participants 2–<5, 5–<12, and ≥12 (i.e., 12–<18 and ≥18) years of age, respectively, with participants receiving the age-appropriate dose at each vaccination. Vaccinations were administered intramuscularly at a study site by a qualified, good clinical practice-trained, vaccine-experienced member of the study staff.

This study is reported here in accordance with TREND (Transparent Reporting of Evaluations with Nonrandomized Designs) guidelines.

### 2.2. Objectives and Endpoints

The primary immunogenicity endpoint was to describe SARS-CoV-2 neutralizing geometric mean titers (GMTs; using the USA_WA/2020 ancestral strain of SARS-CoV-2 [9]) measured 1 month after Dose 3 and Dose 4 in participants without serologic or virologic evidence of previous SARS-CoV-2 infection using previously described assays [30]. Participants who had no serologic or virologic evidence (before the subsequent blood sample collection) of past SARS-CoV-2 infection were defined as having negative N-binding antibody (serum) result at any visit before the subsequent time point, SARS-CoV-2 not detected by a nucleic acid amplification test (nasal swab) until prior vaccination, negative nucleic acid amplification test (nasal swab) result at any unscheduled visit before the subsequent blood sample collection, and no medical history of COVID-19. GMTs were assessed by age group (2–<5, 5–<12, 12–<18, and ≥18 years of age) and disease subset. Exploratory endpoints included the description of GMTs at all immunogenicity blood draws, geometric mean fold rises (GMFRs) from baseline to 1 month after Dose 3 and 1 and 6 months after Dose 4, and the percentage of participants achieving seroresponses at 1 month after Dose 3 and from Dose 4 to 1 month and 6 months after Dose 4 in participants without and in participants with or without evidence of previous SARS-CoV-2 infection.

Primary safety objectives were to describe reactogenicity, including local reactions (pain at the injection site, redness, and swelling) and systemic events (fever, fatigue, headache, chills, vomiting, diarrhea, new or worsened muscle pain, and new or worsened joint pain), and safety, such as adverse events (AEs) by age group and disease subset. Local reactions and systemic events were recorded by the participants or their legal guardians via an electronic diary for 7 days after each dose. AEs from Dose 1 through 1 month after Dose 2, Dose 3 through 1 month after Dose 3, and Dose 4 through 1 month after Dose 4, and serious AEs (SAEs) throughout the study were reported. AEs and SAEs were categorized according to the terms of the Medical Dictionary for Regulatory Activities (MedDRA). AEs of special interest (AESI), including diagnosis of myocarditis or pericarditis and exacerbation of immunocompromising conditions, from Dose 1 through the end of the study (i.e., 6 months after Dose 4) were also reported. The frequency of confirmed cases of COVID-19 occurring among participants was an exploratory objective (see the Supplementary Text for additional details).

### 2.3. Analysis

Because authorization for the emergency use of BNT162b2 in immunocompromised individuals led to enrollment targets (for study participants who were COVID-19 vaccine-naive) not being met [31], all analyses were descriptive. Data were summarized separately by age group and disease subset. The percentage of participants with a seroresponse is reported with two-sided 95% confidence intervals (CIs) computed using the F distribution (Clopper–Pearson). Geometric means were calculated by determining the mean of the logarithmically transformed assay results and then exponentiating the mean to express results on the original scale. GMFRs (ratios of results after vaccination to results before vaccination) were calculated as the mean of the difference between logarithmically transformed assay results and then exponentiating the mean. Associated two-sided 95% CIs were calculated using Student’s *t* distribution for the mean logarithm of the GMTs and GMFRs and exponentiating the confidence limits. Results below the lower limit of quantification (LLOQ) were set to 0.5 × LLOQ in the analysis. Seroresponse was defined as a ≥4-fold rise in neutralizing titer from baseline (before Dose 1); if the baseline measurement was less than the LLOQ, a postvaccination measure of ≥4 times the LLOQ was considered a seroresponse. Immunogenicity and safety populations are defined in Table S1.

## 3. Results

### 3.1. Participants

**Figure 1:**
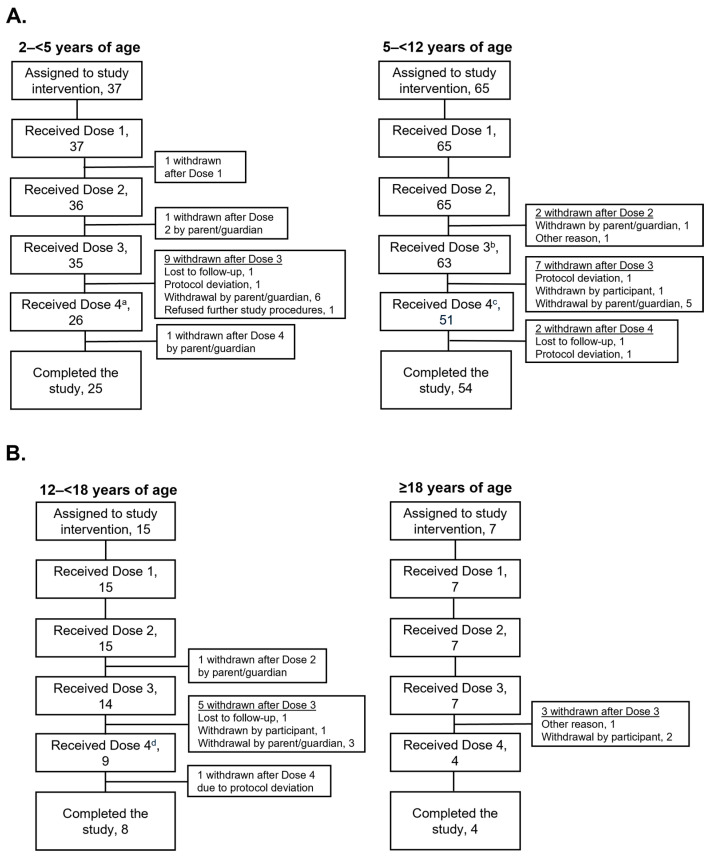
Disposition of participants 2–<5 and 5–<12 years of age (**A**), and 12–<18 and ≥18 years of age (**B**). ^a^ Seven participants received a 10-µg dose level of BNT162b2. ^b^ One participant received a 30-µg dose level of BNT162b2. ^c^ Five participants received a 30-µg dose level of BNT162b2. ^d^ One participant received a 10-µg dose level of BNT162b2.

The disposition of participants by age group is shown in Figure 1. Of 124 participants assigned BNT162b2, 119 received Dose 3 (93.3–100% of participants across age groups) and 90 received Dose 4 (57.1–78.5% of participants across age groups).

Participant demographics and baseline characteristics are shown in Table 1. Across age groups, 53.3 to 60.0% of participants were male. The mean (SD) age at vaccination was 3.3 (0.78) years, 8.4 (1.94) years, 13.1 (1.39) years, and 49.6 (17.81) years for the 2–<5, 5–<12, 12–<18, and ≥18 years of age groups, respectively. Among the age groups, four (10.8%), one (1.5%), four (26.7%), and two (28.6%) participants, respectively, had a positive SARS-CoV-2 status at baseline; of these participants, two (12–<18 years of age) had active SARS-CoV-2 infection (i.e., a positive nucleic acid amplification test) at baseline. No participant had a medical history of COVID-19 at enrollment.

### 3.2. Immunogenicity

**Figure 2:**
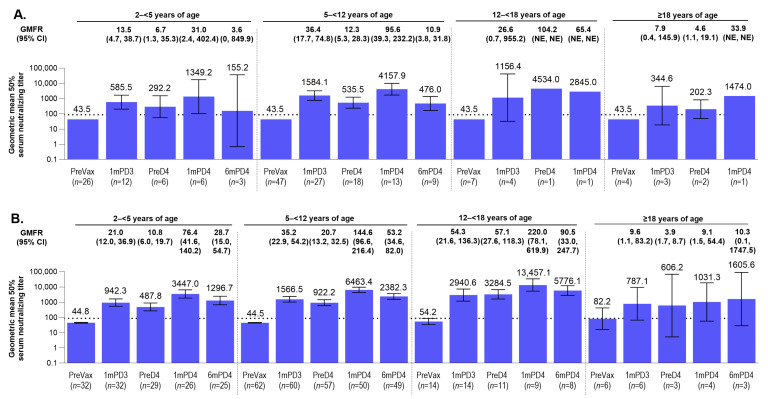
SARS-CoV-2 50% neutralization GMTs and GMFRs from before vaccination in immunocompromised participants without (**A**) and with or without (**B**) evidence of past SARS-CoV-2 infection. Data are for the all-available immunogenicity population. The dotted line represents the LLOQ of the neutralizing assay. GMTs below the LLOQ were set to 0.5 × LLOQ. The error bars are the 95% CIs. CI, confidence interval; GMT, geometric mean titer; GMFR, geometric mean fold ratio; LLOQ, lower limit of quantitation; NE, not estimable; PD, postdose; PreD, predose; PreVax, before vaccination.

Due to low enrollment, which limited the number of participants in the Dose 3 and Dose 4 immunogenicity populations, we primarily present results for the all-available immunogenicity population. Across all age groups of participants without evidence of previous SARS-CoV-2 infection (i.e., seronegative at enrollment), GMTs were numerically higher at 1 month after Dose 3 (GMFR range 7.9–36.4) and 1 month after Dose 4 (GMFR range 31.0–95.6) than before vaccination (Figure 2A). GMTs and GMFRs were also numerically higher at 1 month after Dose 4 than 1 month after Dose 3 (Figure 2A). GMTs among participants without evidence of previous infection were numerically higher at 6 months after Dose 4 in the 2–<5 (GMT, 155.2, GMFR 3.6) and 5–<12 (GMT, 476.0, GMFR 10.9) years of age groups than before vaccination; however, the small number of participants (*n* = 3) in the 2–<5 years of age group limited data interpretability. Due to low enrollment, there was only one participant each in the 6 months after Dose 4 groups for participants in the 12–<18 and ≥18 years of age groups. GMTs and GMFRs for participants without evidence of previous SARS-CoV-2 infection by disease subset are shown in Figure S1.

Similar to participants without evidence of previous SARS-CoV-2 infection, across all age groups of participants with or without evidence of previous SARS-CoV-2 infection, GMTs were higher at 1 month after Dose 3 (GMFR range 9.6–54.3) and 1 month after Dose 4 (GMFR range 9.1–220.0) than before vaccination in each age group (Figure 2B). GMTs were also higher at 1 and 6 months after Dose 4 compared with before Dose 4 across all age groups (Figure 2B). GMFRs from before vaccination to almost all time points were higher in those with or without previous infection than in those without previous infection (Figure 2). Trends in GMT levels and GMFRs among the Dose 3 and Dose 4 evaluable immunogenicity populations (Figure S2) were generally similar to those from the all-available immunogenicity population.

**Figure 3:**
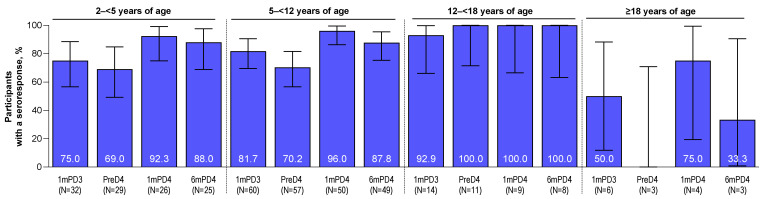
Percentage of participants with or without evidence of infection who achieved a seroresponse among those 2–<5, 5–<12, 12–<18, and ≥18 years of age. Data are for the all-available immunogenicity population. GMTs below the LLOQ were set to 0.5 × LLOQ. The error bars are the 95% CIs. CI, confidence interval; GMT, geometric mean titer; GMFR, geometric mean fold ratio; LLOQ, lower limit of quantitation; NE, not estimable; PD, postdose; PreD, predose; PreVax, before vaccination.

Among participants in the 2–<5, 5–<12, and 12–<18 years of age groups with or without evidence of previous SARS-CoV-2 infection, seroresponse rates ranged from 75 to 92.9% at 1 month after Dose 3, 92.3 to 100% at 1 month after Dose 4, and 87.8 to 100% at 6 months after Dose 4 (Figure 3). The interpretability of the seroresponse data for participants in the ≥18 years of age group was limited due to the small number of participants. Seroresponse data for participants without evidence of previous SARS-CoV-2 infection were also not presented due to the small number of participants in this group, which limited the interpretability of the data.

### 3.3. Safety

#### 3.3.1. Local Reactions and Systemic Events

**Figure 4:**
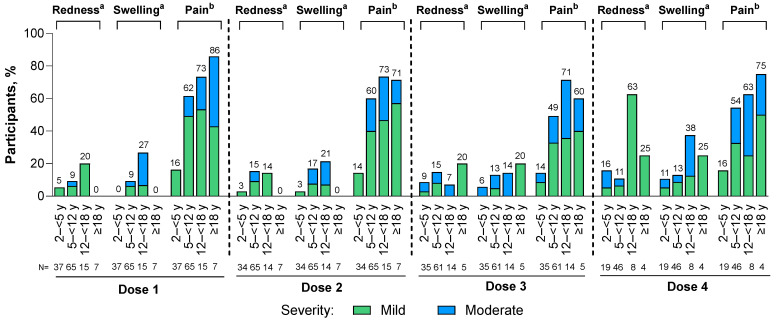
Local reactions occurring within 7 days after each vaccine dose. The numbers above the bars are the percentage of participants with that local reaction overall. ^a^ For participants 2–<12 years of age: mild: 0.5–2.0 cm; moderate: >2.0–7.0 cm; for participants ≥12 years of age: mild: >2.0–5.0 cm; moderate: >5.0–10.0 cm. ^b^ Mild: does not interfere with activity; moderate: interferes with activity.

Across all age groups, local reactions were mild or moderate in severity; no severe or Grade 4 local reactions were reported (Figure 4). Pain at the injection site was the most frequent local reaction and, within each age group, the frequency was similar for each subsequent dose, ranging from 14.3 to 16.2% in participants 2–<5 years of age, 49.2 to 61.5% in participants 5–<12 years of age, 62.5 to 73.3% in participants 12–<18 years of age, and 60.0 to 85.7% in participants ≥18 years of age.

**Figure 5:**
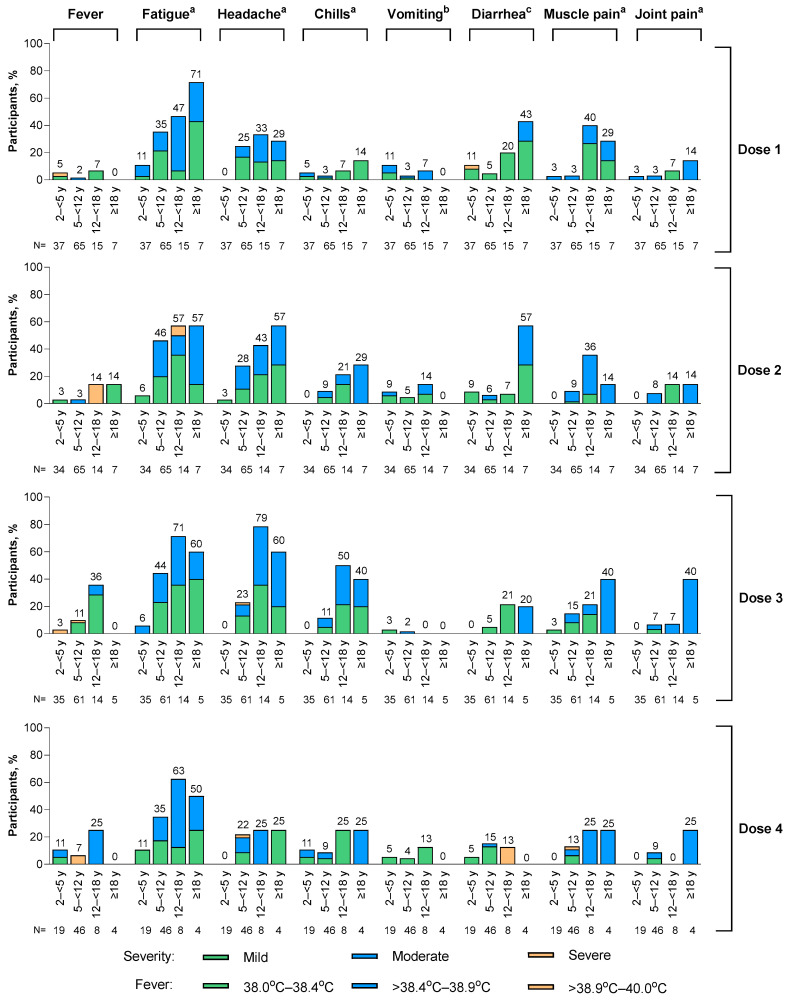
Systemic events occurring within 7 days after each vaccine dose. The numbers above the bars are the percentage of participants with that systemic event overall. ^a^ Mild: does not interfere with activity; moderate: interferes with activity; severe: prevents daily activity. ^b^ Mild: one to two times in 24 h; moderate: more than two times in 24 h; severe: requires intravenous hydration. ^c^ Mild: two to three loose stools in 24 h; moderate: four to five loose stools in 24 h; severe: six or more loose stools in 24 h.

Across all age groups, most systemic events were mild to moderate in severity; no Grade 4 events were reported (Figure 5). Fatigue was the most frequent systemic event, occurring across subsequent doses at frequencies of 5.7 to 10.8% in participants 2–<5 years of age, 34.8 to 46.2% in participants 5–<12 years of age, 46.7 to 71.4% in participants 12–<18 years of age, and 50 to 71.4% in participants ≥18 years of age. Headache was also relatively common in those ≥5 years of age, with 78.6% of participants 12–<18 years of age reporting headache after Dose 3.

#### 3.3.2. Adverse Events

**Figure 6:**
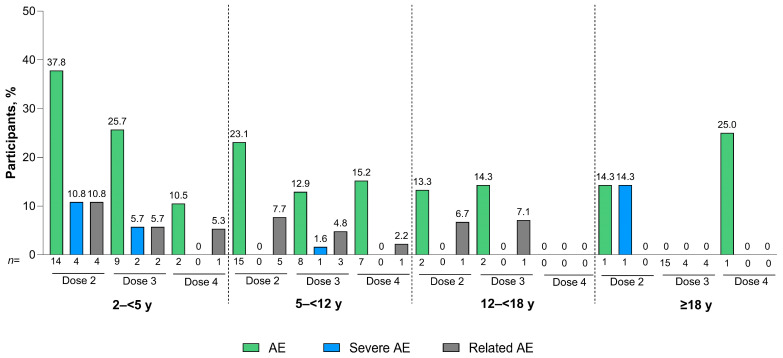
AEs, severe AEs, and vaccine-related AEs by age group and dose. Vaccine-related AEs were assessed by the investigator as related to the investigational product. AE, adverse event; Dose 2, Dose 1 to 1 month after Dose 2; Dose 3, Dose 3 to 1 month after Dose 3; Dose 4, Dose 4 to 1 month after Dose 4.

Frequencies of AEs by age group and vaccine-related AEs by age group and preferred terms are shown in Figure 6 and Table 2. Across most age groups, AEs and vaccine-related AEs tended to be reported more frequently during the period from Dose 1 to 1 month after Dose 2, ranging from 13.3% (2/15) and 6.7%, (1/15), respectively, in participants 12–<18 years of age to 37.8% (14/37) and 10.8% (4/37) in participants 2–<5 years of age (Figure 6). Severe AEs were reported in four (10.8%) and two (5.7%) participants 2–<5 years of age from Dose 1 to 1 month after Dose 2 and from Dose 3 to 1 month after Dose 3, respectively; one severe AE was reported in a participant 5–<12 years of age (from Dose 3 to 1 month after Dose 3) and in a participant ≥18 years of age (from Dose 2 to 1 month after Dose 2). From Dose 1 to 1 month after Dose 2, there were four (10.8%) SAEs in participants 2–<5 years of age, and from Dose 3 to 1 month after Dose 3, there were four (11.4%) SAEs in participants 2–<5 years of age and one (1.6%) SAE in participants 5–<12 years of age. From Dose 1 to the end of the study, SAEs were reported by 11 participants (29.7%) 2–<5 years of age, 11 participants (16.9%) 5–<12 years of age, and two participants (28.6%) ≥18 years of age. No SAEs were related to the vaccine. From Dose 1 to the end of the study, seven participants reported AESIs due to worsening of underlying conditions (Table S2). There were no AEs leading to study withdrawal, no AESIs of myocarditis or pericarditis, and no deaths.

### 3.4. COVID-19 Cases

COVID-19 cases were mostly mild to moderate and were reported in 45 participants (*n* = 3, *n* = 15, *n* = 20, and *n* = 7 between Dose 1 and Dose 2, Dose 2 and Dose 3, Dose 3 and Dose 4, and after Dose 4, respectively). Only one case was severe, occurring in a participant 5–<12 years of age (meeting at least one severe illness criterion: low systolic blood pressure of 80 mmHg); no cases of multisystem inflammatory syndrome were reported.

## 4. Discussion

This descriptive study examined the immunogenicity and safety of a three-dose primary vaccination series with the mRNA BNT162b2 vaccine in immunocompromised participants ≥2 years of age and showed a vaccine-elicited immune response after three doses. When a fourth dose was added, the vaccine-elicited immune response further increased after Dose 4 across all participants and remained elevated at 6 months after Dose 4 in most disease subsets. Immune responses were generally similar across age groups and subsets of immunocompromising conditions. BNT162b2 was safe and tolerable, exhibiting a favorable benefit-risk profile across age groups.

Findings from the current descriptive study are consistent with previous studies evaluating the immunogenicity and efficacy of mRNA COVID-19 vaccines in immunocompromised populations. A prospective study of immunocompromised participants 5–21 years of age, approximately half of whom were SOT recipients who had received two doses of an mRNA COVID-19 vaccine, found that 86.5% had detectable humoral responses after a two-dose series, with significant increases in antibody levels after a third dose [28]. In two studies of immunocompromised adults who received an mRNA COVID-19 vaccine, vaccine efficacy (VE) against COVID-19 hospitalization was higher in immunocompetent versus immunocompromised participants; however, VE in immunocompromised participants was significantly higher after a third dose of the vaccine than after the second dose [32,33]. In a systematic review that included 2838 older immunocompromised adults (the majority were >50 years of age) who had received a COVID-19 vaccine (nearly all mRNA vaccines), seroconversion and antibody titer levels increased after a fourth vaccine dose, regardless of the strength of the serologic response after Dose 3 [27].

This descriptive study did not include a control arm of immunocompetent individuals; therefore, formal statistical comparisons between age groups and comparisons of immunogenicity with healthy individuals could not be made. However, a previous study of healthy children 2–4 years of age without evidence of previous SARS-CoV-2 infection reported a GMFR (95% CI) of 73.3 (66.3–81.1) [10], compared with a GMFR of 13.5 (4.7–38.7) in immunocompromised children 2–<5 years of age in the current study, 1 month after Dose 3 (3-μg dose level) of BNT162b2.

In this study, BNT162b2 was found to be safe and tolerable, supporting results from the OCTAVE-DUO trial, which reported no BNT162b2 vaccine-related SAEs among immunocompromised adults after a third dose of BNT162b2 [26]. A recent systematic review, which included nine predominantly postmarketing surveillance studies of immunocompromised adolescents and young adults, reported that BNT162b2 had an acceptable safety profile and did not cause any severe adverse reactions after a second vaccine dose [34]. Also, in a retrospective cohort study of immunocompromised children vaccinated with BNT162b2, side effects were reported to be mild following vaccination [35]. Most side effects from BNT162b2 were mild and included injection site pain, fatigue, and headache. Considering the small number of participants in some subgroups, the numerical differences in local reactions and systemic events between age groups and disease subsets were not considered clinically meaningful. The relatively high rate of 45 symptomatic, confirmed COVID-19 cases reported is likely due to the study follow-up period being undertaken during the Omicron variant wave at a time of high rates of COVID-19 throughout the population [36], although only one case was classified as severe, with confirmed cases typically occurring after Dose 2 or 3.

At the time the study was conducted, BNT162b2 was available for use in immunocompromised individuals; the aim was to describe safety and immunogenicity in a clinical trial. As a consequence of BNT162b2 being authorized for emergency use in the United States and Mexico, and approved in Germany and Brazil before study initiation [31,37,38,39], the study was limited by the real-world rollout and universal use of BNT162b2, precluding enrollment of the intended number of participants and limiting the interpretability of the data. Due to the small number of participants in the study, the further reduction in participants because of withdrawal from the study also limited the interpretability of the data, including comparisons between age groups. Additionally, the study was initiated before current versions of variant-adapted mRNA vaccines were widely available; however, a recent observational study demonstrated that the bivalent BA.1-adapted BNT162b2 vaccine elicited robust humoral responses in individuals with HIV and kidney transplant recipients [40]. Furthermore, for ethical reasons, the vaccine was made available to all individuals, precluding inclusion of a placebo arm in the study design. Real-world use of BNT162b2 also precluded inclusion of a control arm of immunocompetent individuals.

## 5. Conclusions

This descriptive immunogenicity and safety study demonstrates that BNT162b2 is safe, tolerable, and immunogenic in immunocompromised participants ≥2 years of age. Continued evaluation of the effectiveness and durability of BNT162b2-induced protection remains important for immunocompromised populations.

## Figures and Tables

**Table 1:** Participant Demographics and Baseline Characteristics (Safety Population).

Characteristic	Immunomodulatory Therapy ^a^	Solid Organ Transplant	Stem Cell Transplant	Total
2–<5 years of age, N	9	15	13	37
Sex, *n* (%)				
Male	5 (55.6)	7 (46.7)	10 (76.9)	22 (59.5)
Female	4 (44.4)	8 (53.3)	3 (23.1)	15 (40.5)
Race, *n* (%)				
White	9 (100)	11 (73.3)	12 (92.3)	32 (86.5)
Black	0	2 (13.3)	0	2 (5.4)
Asian	0	1 (6.7)	0	1 (2.7)
Multiracial	0	0	1 (7.7)	1 (2.7)
Not reported	0	1 (6.7)	0	1 (2.7)
Ethnicity, *n* (%)				
Hispanic/Latino	1 (11.1)	1 (6.7)	4 (30.8)	6 (16.2)
Non-Hispanic/non-Latino	8 (88.9)	14 (93.3)	9 (69.2)	31 (83.8)
Age at vaccination, y				
Mean (SD)	3.1 (0.60)	3.6 (0.83)	3.1 (0.76)	3.3 (0.78)
Median (min, max)	3.0 (2, 4)	4.0 (2, 5)	3.0 (2, 4)	3.0 (2, 5)
Baseline SARS-CoV-2 status, *n* (%)				
Positive ^b^	1 (11.1)	3 (20.0)	0	4 (10.8)
Negative ^c^	8 (88.9)	12 (80.0)	9 (69.2)	29 (78.4)
Missing	0	0	4 (30.8)	4 (10.8)
5–<12 years of age, N	19	24	22	65
Sex, *n* (%)				
Male	7 (36.8)	15 (62.5)	17 (77.3)	39 (60.0)
Female	12 (63.2)	9 (37.5)	5 (22.7)	26 (40.0)
Race, *n* (%)				
White	17 (89.5)	21 (87.5)	19 (86.4)	57 (87.7)
Black	1 (5.3)	0	3 (13.6)	4 (6.2)
Asian	0	1 (4.2)	0	1 (1.5)
Multiracial	0	1 (4.2)	0	1 (1.5)
Not reported	1 (5.3)	1 (4.2)	0	2 (3.1)
Ethnicity, *n* (%)				
Hispanic/Latino	6 (31.6)	2 (8.3)	2 (9.1)	10 (15.4)
Non-Hispanic/non-Latino	13 (68.4)	22 (91.7)	19 (86.4)	54 (83.1)
Age at vaccination, y				
Mean (SD)	8.7 (2.16)	8.1 (2.01)	8.5 (1.71)	8.4 (1.94)
Median (min, max)	10.0 (5, 11)	8.0 (5, 11)	8.5 (6, 11)	9.0 (5, 11)
Baseline SARS-CoV-2 status, *n* (%)				
Positive ^b^	1 (5.3)	0	0	1 (1.5)
Negative ^c^	15 (78.9)	17 (70.8)	17 (77.3)	49 (75.4)
Missing	3 (15.8)	7 (29.2)	5 (22.7)	15 (23.1)
12–<18 years of age, N	7	1	7	15
Sex, *n* (%)				
Male	5 (71.4)	1 (100)	2 (28.6)	8 (53.3)
Female	2 (28.6)	0	5 (71.4)	7 (46.7)
Race, *n* (%)				
White	6 (85.7)	1 (100)	7 (100)	14 (93.3)
Asian	1 (14.3)	0	0	1 (6.7)
Ethnicity, *n* (%)				
Hispanic/Latino	3 (42.9)	0	1 (14.3)	4 (26.7)
Non-Hispanic/non-Latino	4 (57.1)	1 (100)	6 (85.7)	11 (73.3)
Age at vaccination, y				
Mean (SD)	13.4 (1.62)	14.0 (-)	12.6 (1.13)	13.1 (1.39)
Median (min, max)	13.0 (12, 16)	14.0 (14, 14)	12.0 (12, 15)	12.0 (12, 16)
Baseline SARS-CoV-2 status, *n* (%)				
Positive ^b^	3 (42.9)	0	1 (14.3)	4 (26.7)
Negative ^c^	1 (14.3)	1 (100)	6 (85.7)	8 (53.3)
Missing	3 (42.9)	0	0	3 (20.0)
Characteristic	Immunomodulatory Therapy ^a^	Non-Small Cell Lung Cancer	Hemodialysis	Total
≥18 years of age, N	5	1	1	7
Sex, *n* (%)				
Male	2 (40.0)	1 (100)	1 (100)	4 (57.1)
Female	3 (60.0)	0	0	3 (42.9)
Race, *n* (%)				
White	1 (20.0)	0	0	1 (14.3)
Black	2 (40.0)	0	0	2 (28.6)
American Indian or Alaska Native	0	1 (100)	0	1 (14.3)
Multiracial	1 (20.0)	0	0	1 (14.3)
Not reported	1 (20.0)	0	1 (100)	2 (28.6)
Ethnicity, *n* (%)				
Hispanic/Latino	2 (40.0)	1 (100)	1 (100)	4 (57.1)
Non-Hispanic/non-Latino	3 (60.0)	0	0	3 (42.9)
Age at vaccination, y				
Mean (SD)	49.0 (20.35)	40.0 (-)	62.0 (-)	49.6 (17.81)
Median (min, max)	39.0 (31, 73)	40.0 (40, 40)	62.0 (62, 62)	40.0 (31, 73)
Baseline SARS-CoV-2 status, *n* (%)				
Positive ^b^	1 (20.0)	1 (100)	0	2 (28.6)
Negative ^c^	4 (80.0)	0	1 (100)	5 (71.4)

^a^ Immunomodulatory therapy was treatment for an autoimmune inflammatory disorder (e.g., inflammatory arthritis, such as rheumatoid arthritis, psoriatic arthritis, and juvenile idiopathic arthritis, and inflammatory bowel disease, such as ulcerative colitis and Crohn’s disease) at a stable dose (i.e., defined as receiving the same dose for ≥3 months (84 days) with no changes in the 28 days before Visit 1). ^b^ Positive N-binding antibody result at Visit 1, positive nucleic acid amplification test result at Visit 1, or medical history of COVID-19. ^c^ Negative N-binding antibody result at Visit 1, negative nucleic acid amplification test result at Visit 1, and no medical history of COVID-19.

**Table 2:** Participants Reporting ≥1 Vaccine-Related ^a^ Adverse Event by Preferred Term and Age Group ^b^ (Safety Population).

Preferred Term, *n* (%) ^c^ [Dose]	2–<5 Years of Age	5–<12 Years of Age	12–<18 Years of Age
Body temperature increased	0	1 (1.6) [Dose 3]	0
Diarrhea	0	1 (2.2) [Dose 4]	0
Dizziness	0	0	1 (6.7) [Dose 2]
Eye inflammation	1 (2.7) [Dose 2]	1 (1.5) [Dose 2]	0
Eye pain	0	1 (1.5) [Dose 2]	0
Gastritis	1 (2.9) [Dose 3]	0	0
Headache	0	1 (1.5) [Dose 2]	1 (6.7) [Dose 2], 1 (7.1) [Dose 3]
Injection site pain	2 (5.4) [Dose 2]	2 (3.1) [Dose 2]	1 (6.7) [Dose 2]
Injection site erythema	0	1 (1.5) [Dose 2]	0
Injection site bruising	0	1 (1.6) [Dose 3]	0
Lymphadenopathy	0	1 (1.6) [Dose 3]	0
Ocular discomfort	0	1 (1.5) [Dose 2]	0
Photophobia	0	1 (1.5) [Dose 2]	0
Purpura	1 (2.7) [Dose 2]	0	0
Rash	0	1 (1.5) [Dose 2]	0
Skin abrasion	1 (2.9) [Dose 3]	0	0
Synovitis	1 (5.3) [Dose 4]	0	0

Dose 2, Dose 1 to 1 month after Dose 2; Dose 3, Dose 3 to 1 month after Dose 3; Dose 4, Dose 4 to 1 month after Dose 4. ^a^ Assessed by the investigator as related to the investigational product. ^b^ No vaccine-related adverse events were reported in participants ≥18 years of age. ^c^ Percentages based on the number of participants who received the dose.

## Data Availability

Upon request, and subject to review, Pfizer will provide the data that support the findings of this study. Subject to certain criteria, conditions and exceptions, Pfizer may also provide access to the related individual de-identified participant data. See https://www.pfizer.com/science/clinical-trials/trial-data-and-results for more information.

## Supplementary Materials

The following supporting information can be downloaded at: https://www.mdpi.com/article/10.3390/vaccines14070602/s1. Supplementary Text: Study Adherence to Ethical Standards; Key Inclusion and Exclusion Criteria; Criteria for Immunocompromised Participants; Determination of Sars-CoV-2–Related Cases; Table S1. Analysis Populations, Table S2. Percentage of Participants Reporting ≥1 Adverse Event of Special Interest from Dose 1 to the End of the Study by Preferred Term (Safety Population); Figure S1. SARS-CoV-2 50% neutralization GMTs (95% CI) and GMFRs (95% CI) from before vaccination in participants without evidence of past SARS-CoV-2 infection who were 2–<5 years of age (A), 5–<12 years of age (B), 12–<18 years of age (C), and ≥18 years of age (D) in the all-available immunogenicity population by disease subset; Figure S2. SARS-CoV-2 50% neutralization GMTs (95% CI) and GMFRs (95% CI) from before vaccination in participants without (A) and with or without evidence of past SARS-CoV-2 infection in the Dose 3 and Dose 4 evaluable immunogenicity population.

## Abbreviations

The following abbreviations are used in this manuscript:

AE	Adverse event
AESI	Adverse event of special interest
CI	Confidence interval
SAE	Serious adverse event
GMFR	Geometric mean fold rise
GMT	Geometric mean titer
HIV	Human immunodeficiency virus
LLOQ	Lower limit of quantitation
MedDRA	Medical Dictionary for Regulatory Activities
N-binding	SARS-CoV-2 nucleoprotein-binding
NE	Not estimable
NAAT	Nucleic acid amplification test
PD	Postdose
PreD	Predose
PreVax	Before vaccination
SOT	Solid organ transplant
TREND	Transparent Reporting of Evaluations with Nonrandomized Designs
VE	Vaccine efficacy
