# Supplementary material for: Safety and Immunogenicity of the BNT162b2 COVID-19 Vaccine in Immunocompromised Participants 2 Years and Older: Results of an Open-Label Phase 2b Study
Source: Vaccines (Basel). 2026 Jul 8;14(7):602. doi: 10.3390/vaccines14070602 (PMC13416987; doi:10.3390/vaccines14070602)
Supplement: Supplementary file 1 [file vaccines-14-00602-s001.zip › vaccines-4269585_Figure S2.pdf]

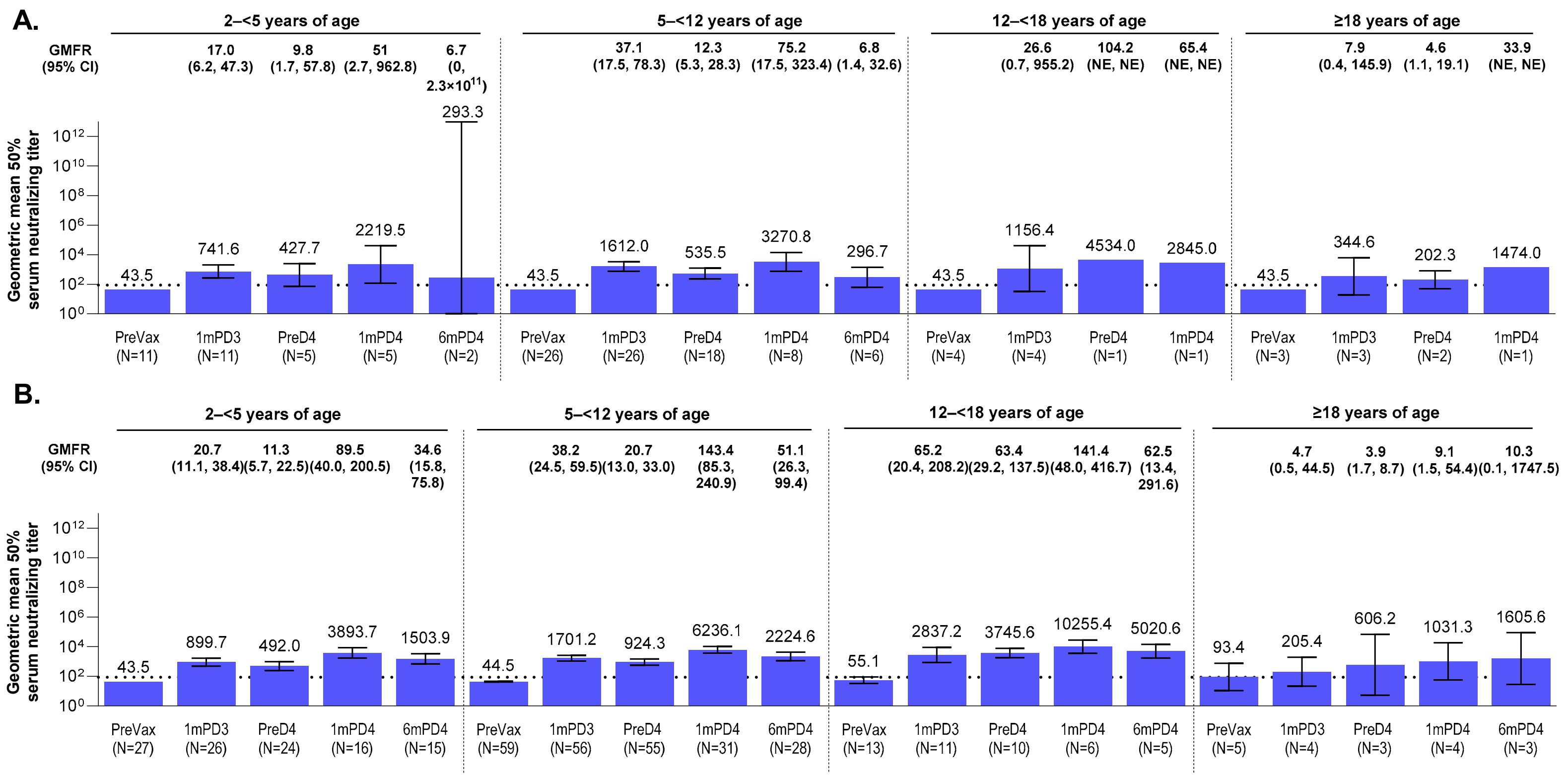

Figure S2. SARS-CoV-2 50% neutralization GMTs (95% CI) and GMFRs (95% CI) from before vaccination in participants without (A) and with or without evidence of past SARS-CoV-2 infection in the Dose 3 and Dose 4 evaluable immunogenicity population. The dotted line represents LLOQ of the neutralizing assay. GMTs below the LLOQ were set to 0.5 × LLOQ. GMT, geometric mean titer; GMFR, geometric mean fold ratio; LLOQ, lower limit of quantitation; NE, not estimable; PD, postdose; PreD, predose; PreVax, before vaccination.
