# Supplementary material for: Safety and Immunogenicity of the BNT162b2 COVID-19 Vaccine in Immunocompromised Participants 2 Years and Older: Results of an Open-Label Phase 2b Study
Source: Vaccines (Basel). 2026 Jul 8;14(7):602. doi: 10.3390/vaccines14070602 (PMC13416987; doi:10.3390/vaccines14070602)
Supplement: Supplementary file 1 [file vaccines-14-00602-s001.zip › vaccines-4269585_Supplementary Text.pdf]

Supplementary Text

Study Adherence to Ethical Standards

The study was conducted in accordance with the ethical principles derived from international guidelines including the Declaration of Helsinki Council and CIOMS International Ethical Guidelines, applicable ICH GCP Guidelines, and other applicable laws and regulations, including privacy laws. The study protocol and other relevant documents were approved by an institutional review board/independent ethics committee before the study was initiated. All participants provided informed consent and assent (as appropriate).

Key Inclusion and Exclusion Criteria

Male and female participants ≥2 years of age at the time of enrollment were eligible. Participants were excluded if they had: 1) a past diagnosis of COVID-19 that was clinical (based on COVID-19 symptoms/signs alone, if a SARS-CoV-2 nucleic acid amplification test result was not available) or microbiological (based on COVID-19 symptoms/signs and a positive SARS-CoV-2 nucleic acid amplification test result) or a past clinical diagnosis of multisystem inflammatory syndrome in children; 2) active graft-vs-host disease, transplant rejection, or posttransplant lymphoproliferative disorder, or treatment for these conditions within 3 months (84 days) before study enrollment; 3) participants <18 years of age whose weight is less than the 5th percentile of age-adjusted ideal body weight (for participants <18 years of age; 4) other medical or psychiatric condition including recent (within the past year) or active suicidal ideation/behavior or laboratory abnormality that may increase the risk of study participation or, in the investigator's judgment, make the participant inappropriate for the study; 5) a history of severe adverse reaction associated with a vaccine or severe allergic reaction to any component of the vaccine; 6) bleeding diathesis or condition associated with prolonged bleeding that would, in the opinion of the investigator, contraindicate intramuscular injection; 7) a previous vaccination with any coronavirus vaccine; 8) ongoing, or a history of, treatment with blood/plasma products or immunoglobulins within 3 months before Dose 1 or planned receipt of these medications before Dose 4; 9) were pregnant or breastfeeding; 10) who may be ineligible because of the number of phlebotomy assessments during this study; 11) or who do not have adequate deltoid muscle mass to allow intramuscular vaccination. Participants who were or had participated in other studies involving the study intervention within 28 days before study entry, had previously participated in other studies involving study intervention containing lipid nanoparticles.

Criteria for Immunocompromised Participants

Eligible participants were immunocompromised as defined by the following criteria: participants ≥2 years who were on active immunomodulator therapy for an autoimmune inflammatory disorder at a

stable dose; participants ≥2 to <18 years of age who had received a solid organ transplant ≥3 months (84 days) before enrollment and with no acute rejection episodes ≥2 months (60 days) before enrollment or who had autologous or allogeneic bone marrow or stem cell transplant ≥6 months (182 days) before enrollment with adequate immune reconstitution for immunization; participants ≥18 years of age with non-small cell lung cancer who were treatment naive or had no chemotherapy 2 weeks before or after dose administration or were receiving checkpoint inhibitor treatment or were receiving targeted therapy and received ≥1 treatment cycle before enrollment; participants ≥18 years of age with chronic lymphocytic leukemia (CLL) with ≥1 of asymptomatic disease and undergoing observation and not receiving treatment for CLL or receiving B-cell inhibitory monoclonal antibody treatment (anti-CD20) and has received ≥3 cycles before enrollment or currently receiving a Bruton tyrosine kinase inhibitor, phosphoinositide 3-kinase (PI3K) inhibitor, or B-cell lymphoma-2 inhibitor; and participants ≥18 years of age currently undergoing maintenance hemodialysis treatment secondary to end-stage renal disease.

Determination of SARS-CoV-2–Related Cases

Confirmed COVID-19 Definition

Confirmed COVID-19 for all participants required the presence of at least 1 of the following symptoms and SARS-CoV-2 NAAT-positive during, or within 4 days before or after, the symptomatic period, either at the central laboratory or at a local testing facility (using an acceptable test):

- Fever;
- New or increased cough;
- New or increased shortness of breath;
- New or increased muscle pain;
- New loss of taste or smell;
- Sore throat;
- Diarrhea;
- Vomiting;
- Inability to eat/poor feeding in participants <5 years of age

Confirmed severe COVID-19

For participants ≥12 years of age the first definition required confirmation of COVID-19 and the presence of ≥1 of the following:

- Admission to an intensive care unit (ICU);
- Death;
- Clinical signs at rest indicative of severe systemic illness (respiratory rate [RR] ≥30 breaths/min, heart rate [HR] ≥125 beats/min, peripheral capillary oxygen saturation [SpO_2] ≤93% on room air at sea level, or partial pressure

of oxygen in arterial blood (PaO_2)/fraction of inspired oxygen (FiO_2) <300 mm Hg);

- Respiratory failure (defined as needing high-flow oxygen, noninvasive ventilation, mechanical ventilation, or extracorporeal membrane oxygenation [ECMO]);
- Evidence of shock (systolic blood pressure [SBP] <90 mm Hg, diastolic blood pressure [DBP] <60 mm Hg, or requiring vasopressors);
- Significant acute renal, hepatic, or neurologic dysfunction.

For participants ≥12 years of age the second definition required confirmation of COVID-19 and the presence of ≥1 of the following:

- Hospitalization;
- Intubation or mechanical ventilation;
- Admission to an ICU;
- Death.

For participants <12 years of age confirmation of COVID-19 and the presence of ≥1 of the following:

- Clinical signs at rest indicative of severe systemic illness (RR and HR and SpO_2 ≤92% on room air or >50% FiO_2 to maintain ≥92%, or PaO_2/FiO_2 <300 mm Hg);
- Respiratory failure (defined as needing high-flow oxygen, including continuous positive airway pressure [CPaP], bilevel positive airway pressure [BiPaP], noninvasive ventilation, mechanical ventilation, or ECMO);
- Evidence of shock or cardiac failure:
- SBP (mm Hg): <70 + (age in years × 2) for age up to 10 years, <90 for age ≥10 years; or requiring vasoactive drugs to maintain blood pressure in the normal range;
- Significant acute renal failure: serum creatinine ≥2 times upper limit of normal (ULN) for age or 2-fold increase in baseline creatinine;
- Significant gastrointestinal/hepatic failure (total bilirubin ≥4 mg/dL or alanine aminotransferase (ALT) 2 times ULN for age;
- Significant neurological dysfunction (Glasgow Coma Scale score ≤11 or acute change in mental status with a decrease in Glasgow Coma Scale score ≥3 points from abnormal baseline);
- Admission to an ICU;
- Death.
