# Supplementary material for: Safety and Immunogenicity of the BNT162b2 COVID-19 Vaccine in Immunocompromised Participants 2 Years and Older: Results of an Open-Label Phase 2b Study
Source: Vaccines (Basel). 2026 Jul 8;14(7):602. doi: 10.3390/vaccines14070602 (PMC13416987; doi:10.3390/vaccines14070602)
Supplement: Supplementary file 1 [file vaccines-14-00602-s001.zip › vaccines-4269585_Table S1.pdf]

Table S1. Analysis Populations

Participants analysis set	Description
Dose 3 evaluable immunogenicity	All eligible participants who received 3 doses of the vaccine, with Dose 2 and Dose 3 received within the predefined window (ie, Dose 2: 19–23 days after Dose 1; Dose 3: 2–25 days after Dose 2), and who had ≥1 valid and determinate immunogenicity result from a blood sample collected within an appropriate window after Dose 3 (ie, 28–35 days after Dose 3), and who had no other important protocol deviations
Dose 4 evaluable immunogenicity	All eligible participants who received 3 doses of the vaccine with Dose 2, Dose 3, and Dose 4 received within the predefined window (ie, Dose 2: 19–23 days after Dose 1; Dose 3: 28–25 days after Dose 2; Dose 4: 91–189 days after Dose 3), and who had ≥1 valid and determinate immunogenicity result from the blood sample collected within an appropriate window after Dose 4 (ie, 28–35 days after Dose 4), and who had no other important protocol deviations
All-available immunogenicity	All participants who received ≥1 dose of the study intervention and who had ≥1 valid and determinate immunogenicity result after vaccination
Safety	All participants who received ≥1 dose of the study intervention
