# Supplementary material for: Safety and Immunogenicity of the BNT162b2 COVID-19 Vaccine in Immunocompromised Participants 2 Years and Older: Results of an Open-Label Phase 2b Study
Source: Vaccines (Basel). 2026 Jul 8;14(7):602. doi: 10.3390/vaccines14070602 (PMC13416987; doi:10.3390/vaccines14070602)
Supplement: Supplementary file 1 [file vaccines-14-00602-s001.zip › vaccines-4269585_Table S2.pdf]

Table S2. Percentage of Participants Reporting ≥1 Adverse Event of Special Interest From Dose 1 to the End of the Study by Preferred Term (Safety Population)

**Preferred Term, *n* (%)**	**Immunomodulatory Therapy [Age Group]**	**Solid Organ Transplant**	**Stem Cell Transplant**
Uveitis	1 (11.1) [2–<5 years of age]	0	0
Rash erythematous	1 (11.1) [2–<5 years of age]	0	0
Tumor necrosis factor receptor-associated periodic syndrome	1 (5.3) [5–<12 years of age]	0	0
Kidney transplant rejection	0	1 (4.2) [5–<12 years of age]	0
Donor specific antibody present	0	1 (4.2) [5–<12 years of age]	0
Crohn’s disease	1 (5.3) [5–<12 years of age]	0	0
Dystrophic calcification	1 (5.3) [5–<12 years of age]	0	0
Dermatomyositis	1 (5.3) [5–<12 years of age]	0	0
